# Moderating Role of Perceived Social Support in the Relationship Between Emotion Regulation and Quality of Life in Chinese Ocean-Going Fishermen

**DOI:** 10.3389/fpsyg.2020.00954

**Published:** 2020-05-26

**Authors:** Yongmei Wu, Sailan Li, Juan Yang

**Affiliations:** ^1^Department of Public Health, Hainan Medical University, Haikou, China; ^2^Hainan Provincial Anning Hospital, Haikou, China; ^3^Key Laboratory of Emergency and Trauma of Ministry of Education, Hainan Medical University, Haikou, China; ^4^Key Laboratory of Brain Science Research & Transformation in Tropical Environment of Hainan Province, Hainan Medical University, Haikou, China

**Keywords:** structural equation modeling, latent moderated structural equations, cognitive reappraisal, expression suppression, WHOQOL-BREF

## Abstract

Perceived social support (PSS) has been shown to be positively related to self-reported quality of life (QoL) as well as to emotion regulation strategy. In the present study, we compared a QoL index between Chinese fishermen (*N* = 507) and local villagers (*N* = 192) and examined whether PSS moderates the relationship between emotion regulation and QoL in our sample of Chinese ocean-going fishermen. Fishermen’s QoL was found to be poorer than that of local villagers. Structural equation modeling (SEM) confirmed that cognitive reappraisal of emotion regulation had a positive predictive effect on QoL, while expression suppression of emotion regulation had a negative predictive effect on QoL. Using, latent moderated structural equations (LMS), we further confirmed that PSS moderates the relationship between emotion regulation and QoL. Simple slope analysis revealed that emotional regulation can predict QoL in a high-PSS context but not in a low-PSS context.

## Introduction

Fishing is a risky occupation with high morbidity and mortality rates (Roberts, 2010; Hjarnoe and Leppin, 2013). Alaskan commercial fishermen were found to have a mortality rate that was 28 times that of Alaskan workers as a whole (Thomas et al., 2001). According to a recent report by the China Fisheries Association, the mortality rate for commercial fishermen in China, at 214 per 100,000, is 2.7-fold higher than the world average of 80 per 100,000 (Jiang et al., 2018). Fishermen spend long periods of time separated from their families while enduring long working hours, hard physical work, and poor sleep conditions (Jeżewska et al., 2012). Their working conditions can lead to hearing impairment, visual impairment, obesity, cardiovascular system diseases, and skin injuries, all of which they are at an elevated risk for relative to the general population (Zeigelboim et al., 2013; Poulsen et al., 2014). Compared to people in other occupations, fishermen are also more likely to smoke, to partake in excessive alcohol consumption, and to develop anxiety and other mental health (Jeżewska et al., 2012). Such findings suggest that the unique physical and mental health challenges experienced by fishermen impact their quality of life (QoL).

Outcomes with respect to the development of physical and mental conditions are influenced by the emotional coping strategies that individuals engage in. According to the process model of emotion regulation (Gross, 2002), particular strategies for modulating one’s emotional responses to stress can have healthful or harmful effects on physical and mental health. For example, the emotion regulation strategy known as *cognitive reappraisal*, which is defined as construing a potentially emotion-eliciting situation in non-emotional terms, can dampen the potential emotional impact of a situation, improve one’s mood, and even promote a better understanding of a stressful situation (Gross, 1998b, 2002). The cognitive reappraisal strategy may reduce depression and anxiety symptoms while improving QoL, well-being, and life satisfaction (Haga et al., 2009; Barberis et al., 2017). Conversely, the emotion regulation strategy known as *expression suppression* is defined as inhibiting ongoing emotion-expressive behavior (Gross, 2002), which is regarded as a potentially maladaptive strategy wherein, after the development of an emotional response that has already been activated and detected, one makes a purposeful effort to mask the external signs of the emotional response (Brans et al., 2013). Emotional suppression has been related to increased physiological arousal (Gross, 1998a) and a reduced capacity for emotion regulation, which may lead to less positive psychology (Phillips et al., 2014; Diefendorff et al., 2018). Based on our systematic review of the literature reporting theoretical and empirical studies of emotional regulation, we posit that perceived social support (PSS) may be an important moderator in the relationship between emotional regulation and QoL. The social background hypothesis of emotional regulation holds that the particular social context in which emotion regulation occurs, including social relationships that build external resources for emotional regulation, may weaken or enhance the influence of emotion regulation strategies on one’s psychological response (Marroquín and Nolen-Hoeksema, 2015). According to this hypothesis, social relationships represent an important aspect of social support because they can help meet individuals’ needs be met, affirm respect for beliefs, attitudes, and values, make people feel understood and cared for (Lyrakos et al., 2016), provide individuals with a sense of security, and become a conduit for increasing the resources individuals need to cope with stress, reduce psychological stress, and achieve personal growth (Hofer, 2016). In a high-PSS context, individuals should feel a sense of respect and understanding that can alleviate emotional imbalance and support emotional stability (Liao and Weng, 2018), thus helping to alleviate negative psychological problems while facilitating positive psychological changes (Collins and Feeney, 2000). However, in a low-PSS context, an individual’s sense of security is tenuous. Consequently, those who have a tendency to suppress their emotional expression are likely to amplify that strategy, which may further aggravate the imbalance between their explicit and internal emotions (Grandey and Alicia, 2000; Extremera and Rey, 2014), thus leading to more negative psychological problems and a greater barrier to positive psychological changes (Giri et al., 2016; Cornelius et al., 2018).

Studies examining the relationship between emotion regulation strategies and QoL seldom take the variable of PSS into consideration. The aim of the present study was to test the hypothesis that PSS may play a positive moderator role in the relationship between emotion regulation and QoL in Chinese commercial fishermen. Toward this aim, we conducted an analysis of emotion regulation strategies and QoL in a cohort of Chinese fishermen as well as in a group consisting of their local villager peers.

## Materials and Methods

### Participants

A group of 507 fishermen (504 males and three females) and a group of 192 local villagers (187 males and five females) were recruited from Tanmen town, Qionghai city, Hainan province, China, during a fishing moratorium. The fishermen participants were recruited through the Hainan Fishery Mutual Protection Association, and the villagers were recruited through the local village committee. There were several inclusion criteria: fishermen participating in ocean-going fishing or local villagers without sea experience; no mental disorders; willingness to participate and provide informed consent for participation; no clinically significant hearing or vision loss; and the ability to understand questionnaire items. All participants provided signed informed consent forms before being enrolled as study participants.

Most of the fishermen (73.4%) had been employed for more than 5 years. The mean age ± standard deviation (SD) of the fishermen and villagers were 36.9 ± 11.3 years (range, 16–66 years) and 35.2 ± 12.9 years (range, 16–65 years), respectively. The vast majority (91.9%) of fishermen had less than a middle school education, and two thirds (66.7%) were smokers. A majority (54.5%) of villagers had less than a middle school education, and about four tenths (41.8%) were smokers. With respect to marital status, 74.4% of fishermen were married, 23.1% were unmarried, and 2.5% divorced or separated, whereas 69.8% of the villagers were married, 27.5% were unmarried, and 2.7% were divorced or separated.

Ethical approval for all study procedures was obtained from the Hainan Medical University Ethics Committee. Trained researchers administered questionnaires to participants.

### Measures

#### QoL

QoL was assessed with the WHO Quality of Life-BREF (WHOQOL-BREF), a 26-item version of the original 100-item source questionnaire. The WHOQOL-BREF contains one item for each of 24 QoL facets, a general QoL item, and a general health item. The items constitute four domains: physical health, psychological health, social relationships, and environment. Item responses ranged from 1 (very dissatisfied/very poor) to 5 (very satisfied/very good), with higher scores indicating a better perceived QoL (Group, 1998). In the present study, we obtained Cronbach’s alpha coefficient of 0.89 for the WHOQOL-BREF, and found that it had acceptable structural validity with the following fitting index values: χ^2^*/*degrees of freedom (*df*) = 2.95, comparative fit index (CFI), Tucker-Lewis index (TLI) = 0.86, and root mean square error of approximation (RMSEA) = 0.06.

#### PSS

PSS was assessed with a Chinese version of the Multidimensional Scale of Perceived Social Support (MSPSS; Zimet et al., 1988), which has been used extensively to measure PSS from family, friends, and significant others. Specifically, we used a 12-item MSPSS that was translated into Simplified Chinese and shown to have good validity and reliability in Chinese subjects (Chou, 2000). Participants were asked to rate each item on a 7-point Likert-type scale ranging from 1 (very strongly disagree) to 7 (very strongly agree). Higher scores represented higher levels of PSS. The reliability coefficients of the dimensions of family support, friend support, and other support were 0.89, 0.88, and 0.89, respectively. The Cronbach’s alpha coefficient of the 12-item MSPSS scale used in the present study was 0.96, and we found that the scale had good structural validity with the following confirmatory factor analysis fitting index values: χ^2^*/df* = 3.91, CFI = 0.95, TLI = 0.93, and RMSEA = 0.07.

#### Emotion Regulation

We assessed participants’ emotion regulation with a validated Chinese version of the revised emotion regulation questionnaire (ERQ), a 10-item, 7-point Likert type (1 = strongly disagree to 7 = strongly agree) self-report instrument designed to assess respondents’ inclinations to regulate their emotions through each of two strategies: cognitive reappraisal and expressive suppression (Gross and John, 2003). Higher scores represent higher emotion regulation levels. In its validation study, the Chinese ERQ used had Cronbach’s alpha coefficients of 0.85 and 0.77 and retest reliabilities of 0.82 and 0.79 for the cognitive reappraisal and expressive suppression strategies, respectively (Wang et al., 2007). In the current study, the Cronbach’s alpha coefficients for the ERQ were 0.91 for cognitive reappraisal, 0.85 for expression suppression, and 0.93 for the whole ERQ. We further found that the ERQ had good structural validity, with the following confirmatory factor analysis fitting index values: χ^2^*/df* = 1.86, CFI = 0.98, TLI = 0.97, and RMSEA = 0.06.

#### Statistical Analysis

SPSS 22.0 and Mplus 7.4 software were used for the data analysis. First, descriptive statistics were carried out to determine the means, SDs, and internal consistency reliabilities for each scale. Independent sample *t*-tests were conducted to compare QoL scores between fishermen and villagers. A correlation analysis was then used to investigate the relationship between emotional regulation strategy use, PSS, and QoL.

Structural equation modeling (SEM) was used to investigate the effects of cognitive reappraisal and expression suppression on QoL. The SEM included a measurement model and a structural model. First, we tested the measurement model according to the recommendations of Anderson and Gerbing (1988). In our direct effect SEM, the measurement model had three potential variables: cognitive reappraisal of emotion regulation; expressive suppression of emotion regulation; and QoL. QoL itself was composed of four dimensions: physical health; mental health; social relations; and environment. In the structural model, we investigated the influence of cognitive reappraisal and expression suppression on QoL.

Finally, on the basis of an SEM-generated direct effect path diagram, we tested the moderating effects of PSS on emotion regulation and QoL using latent moderated structural equations (LMS). A two-step method was applied for assessing the overall fit of our LMS model (Klein and Moosbrugger, 2000). First, CFI, TLI, RMSEA, and χ^2^-values were obtained from the aforementioned SEM. Second, we used a log-likelihood ratio test to compare the relative fit of Model 0 (null model from SEM wherein the interaction is not estimated and therefore assumed to be zero) and Model 1 (alternative model from SEM wherein the interaction is estimated). If the more parsimonious Model 0 fits well and the log-likelihood ratio test result indicates that Model 1 represents a significant fit improvement relative to Model 0, then it can be concluded that Model 1 is also a well-fitted model (Satorra and Bentler, 2010). If the log-likelihood ratio test is not significant, one can only conclude that Model 0 does not result in a significant loss of fit relative to Model 1. There is no way to assess whether the fit of Model 1 is equal to or worse than that of Model 0. The simple slope test to further judge the moderator role of PSS at high and low levels in the specific path of the influence of emotion regulation strategies on QoL. We applied Wen et al. (2004)’s fit index criteria (2004): χ^2^/*df* < 5, CFI and TLI > 0.90, and RMSEA < 0.08.

## Results

### Differential Analysis of QoL Assessment Scores

Ocean-going fishermen had a significantly lower mean WHOQOL-BREF total score than their local villager peers (Table 1). Comparing WHOQOL-BREF domain scores, we found that, relative to the local villagers, the fishermen had significantly lower psychological and environment domain scores but similar physical and social relations domain scores (Table 1).

**TABLE 1:** Inter-group comparisons of mean QHOQOL-BREF scores.

WHOQOL-BREF domain	Mean score ± SD		*T*	*P*
	
	Fishermen	Villagers		
Physical	15.49 ± 2.66	15.77 ± 2.07	–1.30	0.194
Psychological	13.97 ± 3.31	14.65 ± 2.45	–2.60	0.009**
Social relations	15.19 ± 3.57	15.14 ± 2.56	0.21	0.831
Environment	13.10 ± 3.35	14.30 ± 2.70	–4.31	< 0.001***
Total	57.77 ± 11.30	59.86 ± 8.15	–2.35	0.019*

Independent-sample t-tests: *p < 0.05, **p < 0.01, ***p < 0.001.

### Correlations

The mean score data obtained for the MSPSS, QHOQOL-BREF, and ERQ are reported in Table 2 together with Pearson correlation coefficients and *p-*values. PSS among fishermen (*N* = 507), represented by MSPSS scores, was found to correlate positively with both QoL, represented by WHOQOL-BREF total scores, and emotion regulation, represented by ERQ scores. Additionally, QoL and emotion regulation scores were found to correlate with each other.

**TABLE 2:** Descriptive statistics and Pearson correlations among MSPSS, ERQ, and WHOQOL-BREF scores in fishermen (*N* = 507).

Scale score	Mean	*SD*	1	2	3
1. MSPSS (social support)	57.78	19.00	1.00		
2. ERQ (emotion regulation)	40.25	15.66	0.439**	1.00	
3. WHOQOL-BREF (QoL)	57.77	11.30	0.365**	0.175**	1.00

**p < 0.01.

### Direct Effect Analysis

**FIGURE 1:**
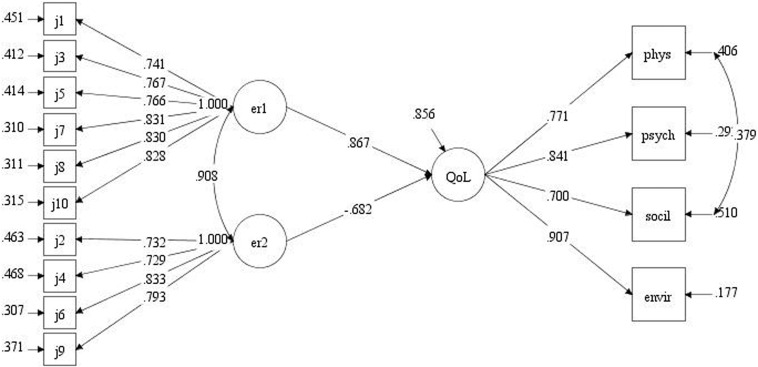
SEM direct effect path diagram. The three main variables examined in our SEM, namely cognitive reappraisal (six components: j1, j3, j5, j7, j8, and j10), and expressive suppression (four components: j2, j4, j6, and j9) are represented by the diagram terms er1 and er2, respectively. The four QoL domains physical health, mental health, social relations, and environment are represented in the diagram as phys, psych, social, and envir, respectively.

We used our SEM measurement model to investigate the influence of the cognitive reappraisal and expression suppression emotion regulation strategies on QoL. To avoid type I errors in our SEM, we first established the status of the correlative relationship between cognitive reappraisal and expressive suppression, which was affirmed to be significant (above). After fitting the model, we found that we needed to modify it slightly. According to the modified index, we found that the physical health and social relation dimensions of QoL were related. We obtained good fitting indexes for the measurement model (χ^2^*/df* = 3, CFI = 0.99, TLI = 0.98, and RMSEA = 0.06), which indicated that it was acceptable. Our standardized structural model results indicated that cognitive reappraisal is a positive predictor of QoL (β = 0.87, *p* < 0.001), expressive suppression is a negative predictor of QoL (β = -0.68, *p* < 0.001), cognitive reappraisal and expressive suppression are significantly correlated (β = 0.90, *p* < 0.001), and the physical health and social relationship dimensions of QoL (as determined by the WHOQOL-BREF) are significantly correlated (β = 0.38, *p* < 0.001). The strengths and directions of relationships among the analyzed variables are summarized in a direct effect path diagram (Figure 1). Overall, the results suggest that more robust cognitive reappraisal can improve QoL, while expressive suppression is not conducive to the improvement of the QoL.

### Moderating Effect Analysis

**FIGURE 2:**
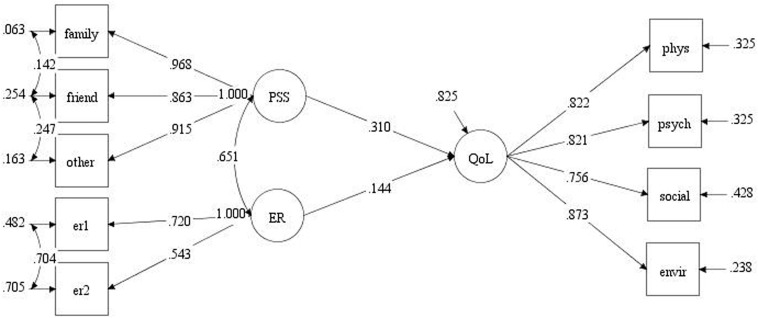
Path diagram without interaction term. ER, emotion regulation; the three PSS domains family support, friend support, and other support are represented in the diagram as family, friend, and other, respectively.

On the basis of an SEM-generated direct effect path diagram, we then used LMS modeling to investigate the moderator effect of PSS on the relationships between emotion regulation strategy and QoL. In our study, Model 0 (Figure 2) fit the data well [χ^2^(21) = 97.55, CFI = 0.97, TLI = 0.95, RMSEA = 0.08]. Log-likelihood ratio testing comparing log-likelihood values between Model 0 and Model 1 yielded a difference value of 22.6. Given that Model 0 and Model 1 had 33 and 34 free parameters, respectively, and the difference in free parameters between them was 1, which represents the *df* value to be used in log-likelihood ratio testing. Hence, using a χ^2^ distribution with a χ^2^-value is 22.6 and *df* of 1 (*p* < 0.001), we found that Model 1 produced a significantly better fit for the data than Model 0.

**FIGURE 3:**
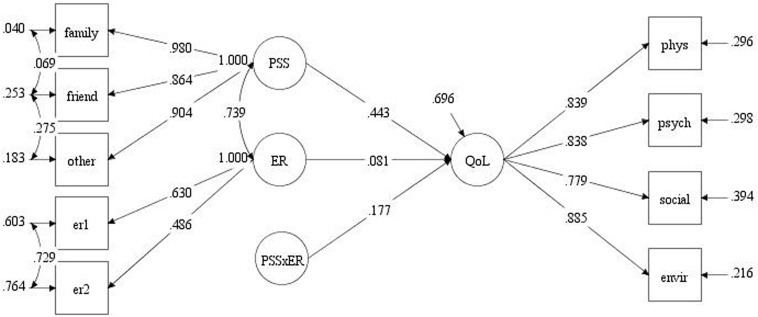
Moderating effect path diagram. Abbreviations: ER, emotion regulation and PSS × ER, PSS-emotion regulation interaction term.

Three regression paths affecting the QoL were set in the structural model, including emotion regulation, PSS, and their interaction items, which are potential variables that could be extracted from the dimensions. Specifically, the latent variable PSS included family, friend, and other dimensions; the friend dimension was related to the family dimension and to the other dimension to reduce type I error risk. Our moderated path analysis indicated the presence of a significant moderating effect of PSS (coefficient, 0.177; *p* = 0.02) on the relationship between emotion regulation and QoL (Figure 3).

**FIGURE 4:**
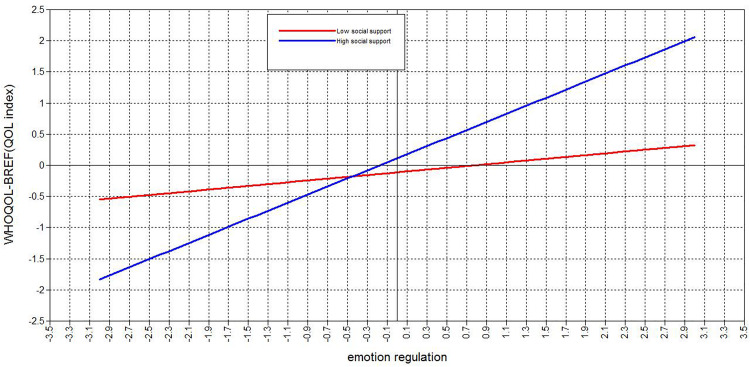
Moderating effect simple slope test results.

Subsequently, standardized simple slope testing showed that in the context of a high PSS level, emotion regulation had a strong positive predictive influence on QoL (β = 0.46, *p* < 0.05). Meanwhile, in the context of a low PSS level, emotion regulation was not a significant predictor of QoL (β = 0.32, *p* = 0.15). Hence, our standardized simple slope testing results suggested that with an increasing level of PSS, emotional regulation becomes a better predictor of QoL (Figure 4).

## Discussion

In the present study, our data demonstrated a moderator role of PSS in the relationship between emotion regulation and QoL in Chinese commercial fishermen. Cognitive reappraisal was found to have a significant positive predictive effect on QoL, whereas expression suppression had a significant negative predictive effect on QoL. These results are consistent with previous studies suggesting that the cognitive reappraisal strategy promotes adaptive psychological changes related to life satisfaction, well-being, and mental health (Brans et al., 2013; Pavani et al., 2016). Conversely, our data indicate that expression suppression can impede emotion regulation, consistent with prior work suggesting that the expression suppression strategy makes it more difficult for people to deal effectively with their emotional responses, which can hinder the development or maintenance of a positive psychological status (Haga et al., 2009). We found that Chinese fishermen had a significantly lower QoL, as measured by the WHOQOL-BREF, than local villagers. The significance in the total score comparison was attributable to the psychological and environment domains, suggesting that a relatively low QoL among fishermen may be related to their harsh working conditions and long-term separation from family (Hjarnoe and Leppin, 2013). Müller et al. (2016) likewise found that the QoL of Brazilian fishermen were lower than that of the general population, with physical health being the most impaired area. In a study of Chinese seafarers, Xiao et al. (2017) found that environmental domain scores were significantly lower than other QoL domain scores, consistent with our supposition that the particular characteristics of the fisherman occupation explain, at least in part, the lower QoL of fishermen compared to the general population.

We found that fishermen’s emotion regulation can improve their QoL in the context of a high PSS level. In this regard, it can be supposed that with a high level of PSS, fishermen can gain a sense of security, belonging, understanding, respect, and help from others (Lyrakos et al., 2016), which would be conducive to increasing their resources for coping with stress (Yalçín, 2011; Hofer, 2016). The amelioration of the negative effects of stress through such resources may thus be helpful for improving QoL. Our finding of no significant predictive effect of PSS on QoL in a low-level PSS setting may be due to individuals having to rely nearly fully on their own cognitive activities to deal with stressful situations and negative emotions. In this circumstance, cognitive reappraisal may become a critically important emotion regulation strategy among fishermen, with those who have a tendency to employ it benefiting from it while others who are more inclined to adopt expression inhibition struggle with conflicts between their inner and outer emotional states (Grandey and Alicia, 2000). Thus, it is possible that the disparate influences of these two emotion regulation strategies may mask one another, leading to an overall non-significant influence of emotion regulation on QoL in a low-PSS context.

The results of our study should be interpreted in the context of the study’s limitations. First, our study involved a limited sample of ocean-going fishermen working out of a single location in Hainan province, China. Larger samples of participants from different cities should be examined. Second, potential self-reporting related response distortions may affect the interpretability of our results. Finally, because we obtained cross-sectional, rather than longitudinal, data, we cannot make conclusions regarding causal relationships between the examined variables.

## Conclusion

The present data showed that Chinese commercial fishermen have a significantly lower QoL than their local peers, mainly due to differences in the psychological and environmental domains of QoL. Furthermore, supporting and expanding upon the process model of emotion regulation (Gross, 2002), we found that the effect of emotion regulation on QoL is dependent upon PSS level. It can be said that these findings provide empirical support for the theoretical concept of positive psychology.

Importantly, these results provide reference information that may be useful in the provision of mental health support to ocean-going workers. More specifically, given the positive and negative impacts of cognitive reappraisal and expression suppression, respectively, on QoL, our findings suggest that those who provide psychological assistance to fishermen should guide them to think actively about stressors and to contemplate the positive outcomes of their voyages while advising them about expressing their emotions in a way that alleviates psychological pressure. Such support may help to promote positive emotional states while improving QoL. Moreover, our finding showing that high PSS can bolster the positive influence of emotion regulation on QoL in fishermen suggests that social and governmental organizations should formulate measures that protect fisherman interests and encourage fishermen’s families and friends to provide more care and support for them.

## Data Availability Statement

This manuscript contains previously unpublished data. The name of the repository and accession number(s) are not available.

## Ethics Statement

The studies involving human participants were reviewed and approved by the Ethics Committee of Hainan Medical University. The patients/participants provided their written informed consent to participate in this study. Written informed consent was obtained from the individual(s) for the publication of any potentially identifiable images or data included in this article.

## Author Contributions

YW ran statistical models and wrote the manuscript. SL collected data. JY oversaw the project, collected data, and contributed financial backing.

## Conflict of Interest

The authors declare that the research was conducted in the absence of any commercial or financial relationships that could be construed as a potential conflict of interest.
